# Performance of Machine Learning Models for Prognosis Prediction in Oral Cavity Squamous Cell Carcinoma: A Systematic Review

**DOI:** 10.3390/cancers18142261

**Published:** 2026-07-14

**Authors:** Sammy Y. Gao, Jonathan M. Hughes, Shaun A. Nguyen, Bryce S. McCaulay, Jason G. Newman

**Affiliations:** Department of Otolaryngology—Head and Neck Surgery, Medical University of South Carolina, Charleston, SC 29425, USA; gaos@musc.edu (S.Y.G.); hughejon@musc.edu (J.M.H.); macaulab@musc.edu (B.S.M.); newmajas@musc.edu (J.G.N.)

**Keywords:** head and neck cancer, oral cavity, squamous cell carcinoma, prognosis, machine learning, overall survival

## Abstract

**Background/Objectives**: Machine learning (ML) models have increasingly been applied to prognostic prediction in oral cavity squamous cell carcinoma (OCSCC), though their performance and methodological quality remain variably reported. This systematic review evaluated contemporary ML-based prognostic models for clinically relevant OCSCC outcomes, with emphasis on independently validated studies. **Methods**: A systematic review was conducted according to PRISMA guidelines using PubMed, Scopus, Cochrane Library, and CINAHL databases through 1 December 2025. Studies evaluating ML or artificial intelligence prognostic models in adult OCSCC patients were included. Outcomes included overall survival, recurrence, disease-free survival, recurrence-free survival, disease-specific survival, cancer-specific survival, progression, and nodal metastasis. Data extraction and risk-of-bias assessment using PROBAST + AI were performed independently by reviewers. **Results**: Forty studies comprising 105,619 patients met inclusion criteria. ML architectures included random forests, support vector machines, gradient boosting methods, neural networks, and deep learning frameworks. Most models incorporated clinical and pathologic variables, while many integrated radiologic, immunologic, or genomic features. For overall survival prediction, independently validated models generally demonstrated AUCs between 0.80 and 0.90. Recurrence prediction models similarly showed favorable discrimination, with most externally validated studies reporting acceptable predictive performance. Additional prognostic endpoints including disease-free survival, progression, and nodal metastasis demonstrated AUCs ranging from 0.70 to 0.90. Common methodological limitations included retrospective design, small sample size, inadequate external validation, and risk of overfitting. **Conclusions**: ML-based prognostic models in OCSCC demonstrate generally favorable predictive performance across survival and recurrence outcomes. However, substantial heterogeneity in methodology and limited external validation continue to restrict clinical implementation. Future work should prioritize prospective multicenter validation, standardized reporting, and reproducible modeling frameworks.

## Simple Summary

Oral cavity squamous cell carcinoma is a common type of oral cancer, and predicting which patients are most likely to experience recurrence, disease progression, or reduced survival remains challenging. In recent years, machine learning has been increasingly used to analyze large amounts of clinical, imaging, pathology, and molecular data to improve prognostic predictions. This systematic review examines published studies that developed machine learning models to predict outcomes in patients with oral cavity squamous cell carcinoma. Across 40 studies involving more than 105,000 patients, many models showed good ability to predict survival and recurrence. However, differences in study design, reporting quality, and validation methods limit confidence in their widespread clinical use. These findings highlight the promise of machine learning for personalized cancer care while emphasizing the need for larger, well-validated studies before routine implementation in clinical practice.

## 1. Introduction

Oral cavity squamous cell carcinoma (OCSCC) is a prevalent malignancy worldwide and remains associated with substantial morbidity and mortality despite advances in surgical and oncologic management [1,2,3]. Prognosis in OCSCC is strongly influenced by factors including locoregional recurrence, nodal metastasis, and disease progression, making accurate prognostic stratification clinically important for treatment selection, surveillance planning, and patient counseling. Historically, prognostic assessment in OCSCC has relied on conventional statistical approaches such as logistic regression, Cox proportional hazards modeling, nomograms, and clinicopathologic staging systems [4,5]. While these methods have demonstrated utility, they are often limited in their ability to account for complex nonlinear interactions among clinical, radiologic, pathologic, and molecular variables [6].

Machine learning (ML) methods offer an alternative framework capable of identifying high-dimensional relationships within large datasets while reducing reliance on predefined assumptions regarding predictor interactions. Unlike traditional statistical models, ML approaches can integrate multimodal data sources and adaptively optimize predictive performance [7,8]. Over the past decade, increasing numbers of studies have evaluated ML-based prognostic models in OCSCC for outcomes including overall survival, recurrence, disease-specific survival, and progression. Reported performance metrics have generally demonstrated favorable discriminatory ability, generating interest in the potential role of ML-assisted prognostic stratification in head and neck oncology.

However, important methodological concerns remain. Many published studies utilize retrospective single-center datasets, limited sample sizes, or internal validation approaches that may overestimate true model performance [9]. In addition, substantial heterogeneity exists regarding predictor selection, model architecture, outcome definitions, and reporting standards [10]. Prior reviews examining artificial intelligence in head and neck oncology have broadly summarized these technologies, but few have focused specifically on prognosis prediction in OCSCC using contemporary ML methodologies and independent validation cohorts [9].

Therefore, the objective of the present systematic review was to evaluate the performance of ML models developed for prognosis prediction in OCSCC, with specific emphasis on independently validated prognostic models and clinically relevant outcomes including overall survival and recurrence. This review additionally aimed to characterize model architectures, input modalities, validation strategies, and methodological quality across the existing literature.

## 2. Materials and Methods

### 2.1. Literature Search

This systematic review was conducted in accordance with Preferred Reporting Items for Systematic Reviews and Meta-Analyses (PRISMA) guidelines (File S1) [11,12]. This systematic review was registered with the Open Science Framework (OSF; Registration ID: JP3FX). A comprehensive literature search of PubMed, Scopus, Cochrane Library, and CINAHL databases was performed from database inception through 1 December 2025. Search strategies incorporated combinations of Medical Subject Headings (MeSH), keywords, and Boolean operators related to oral cavity squamous cell carcinoma, prognosis, survival, recurrence, machine learning, artificial intelligence, deep learning, and predictive modeling. Full search strategies are provided in Supplemental Table S1. Retrieved studies were imported into Covidence systematic review software for screening and management (Veritas Health Innovation Ltd., Melbourne, Australia). Articles published online ahead of print and indexed prior to the search end date were considered eligible for inclusion regardless of their subsequent assignment to a print publication year. The search was limited to English-language publications, and grey literature was not searched.

### 2.2. Eligibility Criteria

Studies were eligible for inclusion if they: (1) included adult patients with OCSCC; (2) evaluated a machine learning or artificial intelligence-based prognostic prediction model; (3) assessed outcomes related to prognosis, including overall survival (OS), recurrence, disease-free survival (DFS), recurrence-free survival (RFS), progression, disease-specific survival (DSS), cancer-specific survival (CSS), or nodal metastasis; and (4) reported performance metrics including area under the receiver operating characteristic curve (AUC), concordance index (C-index), accuracy, sensitivity, or specificity.

Studies utilizing exclusively traditional statistical models without a machine learning component were excluded. Reviews, editorials, conference abstracts without extractable data, and studies not isolating oral cavity primary tumors were also excluded. Independent validation was defined as model evaluation using data not utilized during model training, including separate institutional cohorts, external validation datasets, or predefined held-out test cohorts. Internal validation referred to split-sample validation, cross-validation, or bootstrapping performed within the original development dataset.

### 2.3. Data Extraction

Two reviewers (S.Y.G. and B.S.M.) independently extracted study characteristics, patient demographics, tumor characteristics, model inputs, model algorithms, outcome measures, validation methods, and reported performance metrics. Disagreements were resolved by a third reviewer (S.A.N.). Extracted variables included study country, data source, model architecture, input modality, and prognostic endpoints. Input domains were categorized as clinical, pathologic, radiologic, immunologic, genomic/molecular, or multimodal combinations. Machine learning architectures included tree-based models, support vector machines (SVMs), ensemble learning methods, neural networks, deep learning approaches, and survival-based machine learning frameworks. For studies which evaluated multiple algorithms or varied model inputs, the highest AUC for each outcome was extracted.

### 2.4. Risk of Bias Assessment

Risk of bias was assessed using the PROBAST + AI tool for prediction model development and evaluation studies by S.Y.G. and B.S.M. with disagreements resolved by a third reviewer (S.A.N.) [13]. Domains included participant selection, predictor assessment, outcome determination, and analytical methodology. Particular attention was given to cohort size, handling of missing data, overfitting risk, validation methodology, and transparency of model reporting. Studies were categorized as low, high, or unclear risk of bias.

### 2.5. Statistical Analysis

Study, patient, and clinicopathologic characteristics were summarized descriptively because of substantial heterogeneity and inconsistent reporting across included studies. Continuous variables are presented as the median reported study-level value and range, while categorical variables are presented as the median reported study-level proportion and range among studies reporting that characteristic. Performance metrics for machine learning models, including AUC and C-index, were summarized descriptively in tabular and graphical form rather than quantitatively pooled because of substantial heterogeneity in model architecture, predictor selection, outcome definitions, validation methodology, and incomplete reporting of variance estimates. Definitions of these performance metrics are summarized in Supplementary Figure S1. As a qualitative sensitivity assessment, reported model performance was additionally examined according to validation strategy (independent versus internal validation) and overall PROBAST + AI risk-of-bias classification to evaluate the robustness of conclusions with respect to methodological quality. All statistics were performed in R version 4.6.0 (R Core Team).

## 3. Results

### 3.1. Included Studies

**Figure 1:**
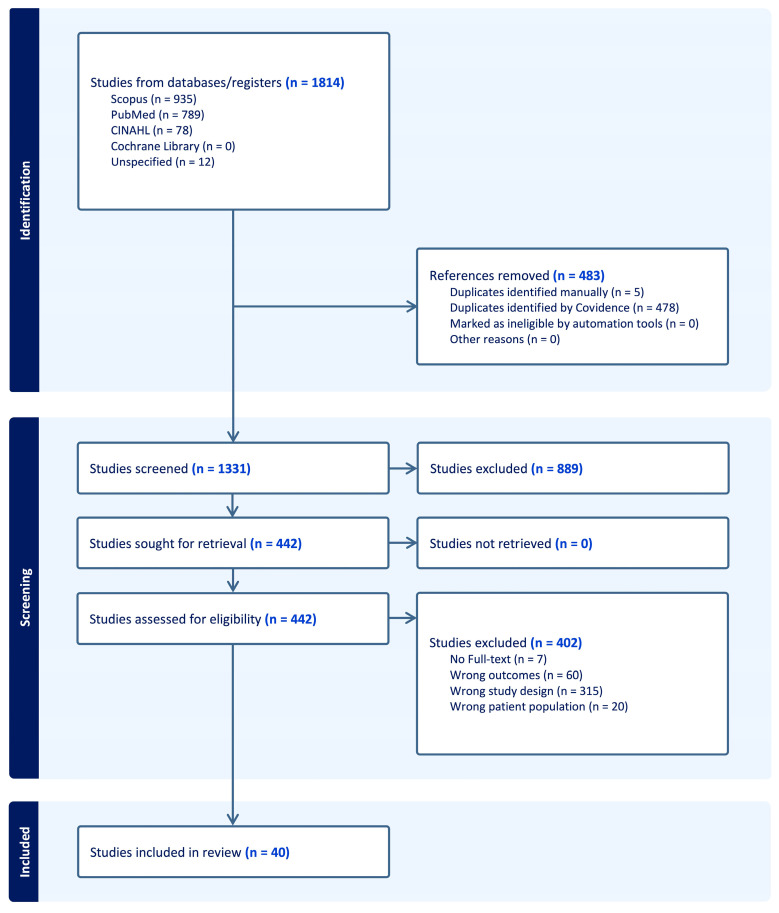
PRISMA Flow Diagram. Copyright statement: this PRISMA diagram contains public sector information licensed under the Open Government License v3.0 [11].

A total of 40 studies met inclusion criteria, encompassing 105,619 patients with OCSCC (Figure 1; Supplemental Table S2). Risk of bias assessed using PROBAST + AI was generally low in the participant, predictor, and outcome domains but more variable in the analysis domain, where concerns frequently related to small sample size, limited outcome events, incomplete reporting or handling of missing data, and lack of robust validation (Supplemental Tables S3 and S4). Overall, studies utilizing larger multicenter or registry-based cohorts with independent validation exhibited lower overall risk of bias and more plausible performance estimates, whereas small, single-center studies employing complex deep learning or multimodal architectures were more likely to report near-perfect discrimination suggestive of optimism bias.

Most investigations employed retrospective cohort designs using registry-based datasets, single-institution cohorts, or multi-institutional databases drawn from North America, Europe, and Asia, including the United States, China, Taiwan, Japan, South Korea, and several European countries. Larger sample sizes were typically observed in registry and multicenter datasets, whereas radiomic and genomic investigations more commonly relied on smaller institutional cohorts.

Across included studies, the median reported patient age was 61 years (range, 50.0–65.3 years; Table 1), and the median proportion of male patients was 68.6% (range, 47.8–92.6%). The tongue was the most frequently represented primary tumor subsite, with a median study-level proportion of 67.6% (range, 33.9–100.0%) among studies reporting tumor location. Adverse histopathologic features, including perineural invasion, lymphovascular invasion, bone invasion, and extranodal extension, were variably reported across studies (Table 1). Most prognostic models incorporated clinical and pathologic covariates, with an increasing proportion of studies integrating radiologic, immunologic, genomic, and multimodal inputs in more recent publications.

A broad range of machine learning architectures were evaluated, including random forests, decision trees, support vector machines (SVMs), gradient boosting methods, neural networks, deep learning frameworks, survival forests, and ensemble learning models with traditional regression-based or nomogram-based approaches.

### 3.2. Overall Survival Prediction

**Figure 2:**
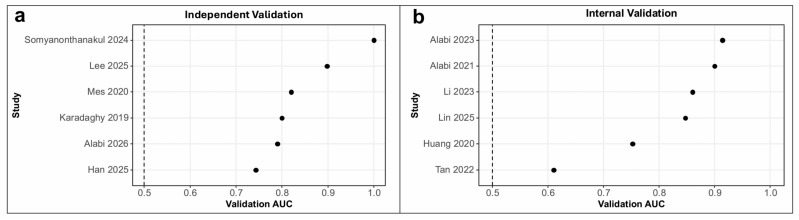
(**a**) [6,14,15,16,17,18], (**b**) [23,24,25,26,27,28]. Independent and Internal Validation AUCs of Machine Learning Models for Overall Survival Prediction. Dot plots include only studies reporting AUC values for the corresponding outcome. Therefore, the number of studies displayed in the figures may differ from the total number of studies included in the outcome-specific tables.

Overall survival (OS) was the most frequently evaluated endpoint, with model performance reported in 20 studies (Table 2, Figure 2). Reported AUC values for OS prediction ranged from 0.60 to 1.00, while C-indices typically varied between 0.70 and 1.00. Among models with independent validation, most AUCs clustered between 0.80 and 0.90, indicating acceptable-to-strong discriminatory ability for OS. Few studies reported calibration measures, decision-curve analyses, or direct comparisons with established prognostic systems.

Commonly used architectures for OS prediction included ensemble learning approaches, random forests, SVMs, and deep neural network-based survival models. Input features most frequently combined clinical and pathologic variables, although several independently validated models additionally integrated radiologic imaging features, immune signatures, or genomic profiles. Notably, independently validated OS models often demonstrated performance metrics comparable to internally validated algorithms, whereas extreme near-perfect AUC values were largely confined to smaller, internally validated datasets, consistent with overfitting and optimism bias.

### 3.3. Recurrence Prediction

**Figure 3:**
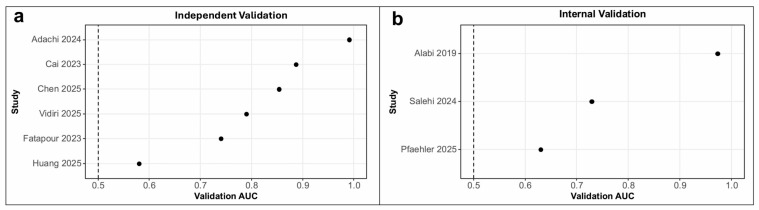
(**a**) [32,33,34,35,36,37], (**b**) [40,41,42]. Independent and Internal Validation AUCs of Machine Learning Models for Recurrence Prediction. Dot plots include only studies reporting AUC values for the corresponding outcome. Therefore, the number of studies displayed in the figures may differ from the total number of studies included in the outcome-specific tables.

Recurrence outcomes, including locoregional recurrence and composite recurrence endpoints, were evaluated in 11 studies (Table 3, Figure 3). Across these models, reported AUC values ranged from 0.60 to 1.00, with most independently validated recurrence models demonstrating AUC values within acceptable prognostic discriminatory thresholds (approximately ≥0.70). Frequently employed model architectures for recurrence prediction included support vector machines, random forests, multilayer perceptrons, gradient boosting methods, and XGBoost-based algorithms. Clinical and pathologic variables formed the core predictor set, while several studies augmented these features with radiologic radiomics or immunologic markers. Independently validated recurrence models generally maintained performance metrics similarly to internally validated counterparts, suggesting potential generalizability despite variation in predictor selection and modeling strategies.

### 3.4. Additional Prognostic Outcomes

Several studies developed machine learning models for additional prognostic endpoints, including (DFS), recurrence-free survival (RFS), disease-specific survival (DSS), cancer-specific survival (CSS), progression, and nodal metastasis (Table 4). Across these outcomes, AUC values typically ranged from 0.70 and 0.90, indicating moderate-to-good discriminatory ability for most models. These analyses utilized diverse algorithms such as random survival forests, decision trees, naïve Bayes classifiers, k-nearest neighbors, deep learning architectures, and radiomic convolutional neural networks. Predictor sets varied widely but generally comprised combinations of clinical staging variables, histopathologic features, imaging-derived radiomics, immunologic signatures, and genomic or transcriptomic markers. Multimodal models often yielded the highest reported performance metrics, although direct comparison across studies was limited by differences in outcome definitions, feature engineering pipelines, and validation strategies.

## 4. Discussion

This systematic review evaluated machine learning models developed for prognosis prediction in oral cavity squamous cell carcinoma and found that ML-based approaches generally demonstrated favorable discriminatory ability across multiple clinically relevant outcomes, including overall survival, recurrence, disease-free survival, and disease-specific survival. Independently validated models, particularly those derived from larger multicenter or registry-based cohorts with lower overall risk of bias, most often reported AUCs in the approximate range of 0.70–0.90, supporting the potential utility of ML-assisted prognostic stratification in OCSCC. AUC was emphasized in this review because it was the most consistently reported performance metric across studies and therefore allowed the broadest comparison of model discrimination. However, AUC alone does not assess calibration, clinical utility, or the impact of model-guided decision making, and should not be interpreted as a comprehensive measure of model quality. Findings remained robust when examined qualitatively according to validation strategy and PROBAST + AI risk-of-bias classification, with extreme near-perfect AUC values largely confined to small, internally validated, higher-risk-of-bias studies suggestive of optimism bias.

Across the literature, a wide range of machine learning architectures were utilized, including ensemble learning methods, random forests, SVMs, gradient boosting approaches, neural networks, and deep learning-based survival frameworks. Most models incorporated multimodal combinations of clinical, pathologic, radiologic, immunologic, or genomic variables, reflecting the increasing emphasis on integrated prognostic modeling in oncology. Notably, while multimodal models frequently demonstrated the highest performance, many relied on radiomic, genomic, immunologic, or digital pathology features that lack standardized acquisition and analytic frameworks. This may limit reproducibility and external generalizability.

These findings are broadly consistent with prior systematic reviews and meta-analyses examining artificial intelligence and prediction models in head and neck cancer, which have similarly reported strong apparent discrimination alongside substantial methodological heterogeneity [8,51]. Adeoye et al. identified promising performance of machine learning models for oral cavity cancer outcomes but highlighted limitations related to heterogeneous methodologies, small datasets, and limited validation [52]. Similar conclusions were reached by subsequent reviews, which emphasized the growing complexity of machine learning approaches alongside persistent concerns regarding reproducibility, external validation, and clinical implementation [8,10,51,53]. Our findings largely corroborate these observations. However, compared with earlier reviews, the current study includes a substantially larger and more contemporary body of evidence, captures recent advances in radiomic, genomic, immunologic, and multimodal prediction models, and applies the PROBAST + AI framework to systematically assess methodological quality and risk of bias. Furthermore, by differentiating independently validated models from internally validated models, our review provides additional insight into model generalizability and identifies a subset of lower-risk-of-bias models with greater potential for clinical translation.

An important observation was that models trained and evaluated using larger multicenter cohorts with independent validation tended to demonstrate more modest but clinically plausible performance estimates, while models developed in small single-institution datasets occasionally reported near-perfect AUCs. This pattern mirrors concerns raised in broader oncology ML research, where overly optimistic internal validation results may reflect overfitting, data leakage, or inadequate separation of training and test sets rather than true generalizability. Accordingly, future efforts should prioritize rigorous external validation of existing models rather than continued development of internally validated algorithms.

Additionally, outcome definitions, predictor selection, and validation methodology were also heterogeneous across studies, limiting direct comparability of results. Some models focused exclusively on conventional clinicopathologic variables, while others incorporated radiomics, digital pathology, immunologic signatures, or genomic data. Although multimodal models frequently achieved high discrimination, their reliance on non-routine imaging or molecular pipelines, variable feature extraction workflows, and complex preprocessing may constrain reproducibility and implementation outside specialized centers. In addition, relatively few studies reported calibration, decision-curve analysis, or clinical utility metrics, which are essential for understanding the practical impact of prognostic models beyond discrimination alone.

From a clinical standpoint, the actionable implications of the current literature remain limited. Although several ML models demonstrated acceptable discrimination for survival, recurrence, and nodal metastasis prediction, few studies evaluated whether model outputs would meaningfully alter treatment selection, adjuvant therapy escalation or de-escalation, surveillance intensity, or patient counseling beyond existing clinicopathologic risk stratification. In addition, relatively few models were directly compared with standard-of-care prognostic systems such as AJCC TNM staging, established nomograms, or conventional Cox-based models. Where comparisons were performed, ML models generally showed comparable rather than clearly superior performance. Therefore, the available evidence does not yet demonstrate consistent incremental clinical utility of ML over established prognostic frameworks. Future studies should explicitly evaluate whether ML-assisted risk stratification improves decision-making, calibration, net benefit, and downstream patient outcomes when compared with current standard prognostic systems.

An additional and important observation from this review was the near-complete absence of calibration assessment and decision-curve analysis among included studies. Although discrimination metrics such as AUC and C-index were frequently reported, these measures alone do not establish whether predicted risks are well calibrated or whether model-guided decisions would improve clinical outcomes. Calibration and decision-curve analyses provide complementary information regarding model reliability and clinical utility and are increasingly recognized as essential components of prediction model evaluation. Their limited use represents a significant methodological and reporting gap within the current OCSCC machine learning literature. Future studies should routinely incorporate and report these metrics to improve transparency, facilitate model comparison, and support eventual clinical implementation.

This review has several limitations. First, substantial heterogeneity in study design, predictor selection, outcome definitions, and reporting of performance metrics precluded formal quantitative meta-analysis of AUCs or C-indices and necessitated a primarily descriptive synthesis of prognostic performance. Additionally, extracting the highest-performing model from each study may introduce optimism bias and overestimate the reported predictive performance across the literature. Although studies were stratified according to the clinical outcome being predicted, further subgroup analyses based on model architecture, predictor modality, or validation approach were not feasible because of substantial overlap between categories, inconsistent reporting, and the limited number of studies within many potential subgroups. In addition, while AUC quantifies a model’s ability to distinguish between patients who do and do not experience an outcome, it does not assess calibration, clinical utility, or whether risk predictions would meaningfully influence management decisions. Consequently, high AUC values should not be interpreted as evidence of clinical usefulness or superiority over existing prognostic tools. Second, incomplete reporting of variance estimates, missing data handling, and potential cohort overlap limited assessment of methodological rigor in some studies and may have biased risk-of-bias judgments. Third, most included studies were retrospective and developed models in single-institution or regional datasets, which may limit generalizability to other practice settings. Fourth, publication bias is possible, as studies reporting strong model performance may be more likely to be published, while negative or inconclusive ML models may remain underreported. Finally, although we attempted prospective registration through PROSPERO, no registration number was issued because PROSPERO does not currently accept systematic reviews focused on diagnostic test accuracy and related prediction model methodologies. This should be considered when interpreting the findings.

Despite these limitations, the present review highlights several priorities for future work. Prospective, multicenter studies with prespecified analysis plans and independent external validation are needed to generate more reliable estimates of ML model performance for OCSCC prognosis. Adoption of standardized reporting frameworks tailored to prediction models using artificial intelligence, such as TRIPOD-AI and PROBAST + AI, will be critical to improve transparency, facilitate reproducibility, and allow robust comparative appraisal of competing models. Future studies should routinely report both discrimination and calibration, incorporate decision-analytic measures of clinical utility, and, where feasible, provide open-source code, model parameters, and harmonized feature definitions to enable independent validation and updating. Ultimately, integration of rigorously validated ML models into clinical decision support will require not only technical performance but also consideration of workflow integration, interpretability, and real-world impact on treatment decisions and patient outcomes.

## 5. Conclusions

The present review provides a focused synthesis of machine learning prognostic modeling in OCSCC and demonstrates that contemporary ML models, particularly multimodal approaches integrating clinical, pathologic, radiologic, and molecular data, generally show favorable prognostic performance across survival and recurrence outcomes. However, substantial heterogeneity in methodology, reporting quality, and validation practices continues to limit generalizability and clinical implementation. Future studies should prioritize prospective multicenter validation, transparent reporting standards, calibration assessment, and reproducible modeling pipelines.

## Figures and Tables

**Table 1:** Demographics.

Characteristic	Number of Studies	Number of Patients	Median	Range
Age	17	105,619	61.1	50–65.3
Male sex	28	73,142	68.6%	47.8–92.6%
White ethnicity	6	30,767	86.6%	78.5–90.4%
Black ethnicity	9	64,710	7.1%	3.5–12.1%
*Site*				
Tongue	24	72,311	67.6%	33.9–100%
Gingiva/gum	12	53,249	19.6%	6.3–55.7%
Buccal mucosa	10	43,730	16.9%	4–64.1%
Floor of mouth	12	46,382	10.3%	0.6–42.2%
Retromolar	8	45,282	3.2%	2.6–13.7%
Hard palate	10	52,984	2.3%	1.6–15.7%
Lips	7	49,467	11.5%	0.5–26.8%
PNI	12	3716	23.3%	8.2–73.8%
LVI	11	3405	17.2%	4–73.8%
BNI	4	1210	22.6%	9.6–31.7%
ECE	10	3095	16.3%	7.6–76.2%
*TNM Staging*				
Tumor (T)				
1	21	28,712	35.8%	0–98.2%
2	21	28,486	33.0%	0–62.7%
3	20	28,315	14.9%	0.6–100%
4	17	27,858	24.10%	0–55.9%
Nodal Status (N)				
0	18	20,833	59.30%	26.1–100%
1	18	28,020	15.60%	7.7–44.9%
2	19	28,070	23.10%	0–76%
3	18	28,020	2.20%	0–16.9%
Metastasis (M)				
0	8	18,520	98.60%	35.7–100%
1	10	25,539	0.40%	0–4.9%

Values are summarized at the study level rather than pooled across all patients. For percentage variables, the median represents the median study-level proportion among studies reporting that characteristic, and the range represents the minimum and maximum reported study-level proportions. For example, the median tongue primary site proportion of 67.6% indicates that among the 24 studies reporting tongue involvement, the median study contained 67.6% tongue tumors. Because reporting varied across studies and many cohorts were restricted to specific oral cavity subsites, percentages are not derived from a common denominator and should not be summed across categories. Similarly, TNM stage percentages represent study-level medians and should not be expected to sum to 100%.

**Table 2:** Performance of Machine Learning Models for Overall Survival Prediction.

Study	Input Type	Model Algorithm	C-Index	AUC	Accuracy (%)	Sensitivity (%)	Specificity (%)
*Independent Validation*							
Somyanonthanakul 2024 [14]	Clinical, Pathologic	Fuzzy Deep Learning		1.00	97.0	90.0	97.0
Lee 2025 [15]	Clinical, Pathologic, Radiologic	Random Forest		0.89		86.0	73.8
Alabi 2026 [16]	Clinical	Voting ensemble		0.79	70.4	68.0	73.0
Karadaghy 2019 [6]	Clinical, Pathologic	Decision Forest		0.80	71.0	68.0	
Mes 2020 [17]	Radiologic, Clinical	Combined radiomic + clinical Cox model		0.82			
Han 2025 [18]	Genomic/Molecular	Elastic Net		0.74			
Deepali 2025 [19]	Clinical, Genomic/Molecular	DeepOmicsSurv	1.00				
Diao 2021 [20]	Immunologic	SVM	0.71				
Kim 2019 [21]	Pathologic	DeepSurv	0.78				
Peng 2022 [22]	Clinical	Random forest	0.80				
*Internal Validation*							
Alabi 2023 [23]	Clinical	Extreme random tree		0.91	86.0	80.0	90.0
Alabi 2021 [24]	Clinical	Boosted Decision Tree		0.90	83.1	85.0	
Li 2023 [25]	Clinical, Immunologic	Light Gradient Boosting Machine		0.86			
Huang 2020 [26]	Genomic/Molecular, Immunologic	Random forest + SVM		0.85			
Lin 2025 [27]	Clinical	DeepSurv-based deep neural network	0.78	0.75		93.1	56.9
Tan 2022 [28]	Clinical	Random Forest classifier		0.61	62.0	59.0	
Adeoye 2022 [29]	Clinical, Pathologic	DeepSurv	0.77				
Nezamabadi Farahani 2024 [30]	Genomic/Molecular	Particle Swarm Optimization + SVM	0.97				
Rosado 2013 [31]	Clinical, Genomic/Molecular	SVM			98.6		
Vollmer 2024 [7]	Clinical, Pathologic, Radiologic, Genomic/Molecular	Random Survival Forest	0.83				

**Table 3:** Performance of Machine Learning Models for Recurrence Prediction.

Study	Input Type	Model Algorithm	C-Index	AUC	Accuracy (%)	Sensitivity (%)	Specificity (%)
*Independent Validation*							
Adachi 2024 [32]	Digital Pathology, Pathologic	CLAM + SVM		0.99	94.9		
Cai 2023 [33]	Clinical, Pathologic, Immunologic	Multilayer perceptron		0.89	83.5	74.0	
Chen 2025 [34]	Genomic/Molecular, Clinical	Random Forest		0.85			
Vidiri 2025 [35]	Clinical, Pathologic	Support Vector Machine		0.79	67.0	67.0	50.0
Fatapour 2023 [36]	Clinical	Gradient Boosting Machine		0.74	80.0	82.8	
Huang 2025 [37]	Clinical, Pathologic	XGBoost		0.58	57.9	57.1	
*Internal Validation*							
Alabi 2020 [38]	Clinical, Pathologic	Boosted Decision Tree			81.0	79.0	83.0
Bourdillon 2023 [39]	Clinical, Pathologic	Decision tree classifier			80.8	80.8	
Alabi 2019 [40]	Clinical, Pathologic	Artificial neural network		0.97	88.2	71.2	98.9
Salehi 2024 [41]	Clinical, Pathologic, Radiologic	Random Forest		0.73	71.2	58.3	78.6
Pfaehler 2025 [42]	Radiologic	Random Forest		0.63	70.0	76.0	50.0

**Table 4:** Performance of Machine Learning Models for Prediction of Metrics of Prognosis.

Study	Input Type	Model Algorithm	Validation Type	C-Index	AUC	Accuracy (%)	Sensitivity (%)	Specificity (%)
*DFS/RFS*								
Alkhadar 2021 [43]	Clinical, Pathologic	Decision tree classifier	Independent		0.77	76.0	81.0	72.0
Mes 2020 [17]	Radiologic, Clinical	Combined radiomic + clinical Cox model	Independent		0.76			
Diao 2021 [20]	Immunologic	SVM	Independent	0.68				
Fujima 2020 [44]	Radiologic	ResNet-101 deep learning classifier	Independent			80.0	80.0	80.0
Tseng 2015 [45]	Clinical, Pathologic	Decision tree	Internal			95.8		
*Progression*								
Mei 2025 [46]	Clinical, Pathologic, Immunologic	K Neighbors Classifier	Independent		0.69	74.5	47.1	80.7
Chu 2020 [47]	Clinical, Pathologic	K-nearest neighbours	Internal		0.71	69.4	35.1	85.6
*Nodal Metastasis*								
Csűry 2024 [48]	Pathologic, Clinical	Naïve Bayes	Independent		0.83			
Pfaehler 2025 [42]	Radiologic	Random Forest	Internal		0.64	67.0	70.0	58.0
*DSS/CSS*								
Csűry 2024 [48]	Pathologic, Clinical	Naïve Bayes	Independent		0.85		77.0	
Peng 2022 [22]	Clinical	Random Forest	Independent	0.84				
Lu 2017 [49]	Pathologic	Quadratic Discriminant Analysis	Internal		0.87	88.0	78.0	93.0
Wang 2023 [50]	Clinical	Random Survival Forest	Internal		0.80			
Adeoye 2022 [29]	Clinical, Pathologic	DeepSurv	Internal	0.89				
Tseng 2015 [45]	Clinical, Pathologic	Decision tree	Internal			81.7		

## Data Availability

No new datasets were generated during this study. The data supporting the findings of this systematic review are available from the corresponding author upon reasonable request and can also be obtained from the original studies cited in the manuscript.

## Supplementary Materials

The following supporting information can be downloaded at: https://www.mdpi.com/article/10.3390/cancers18142261/s1, Figure S1: Definitions of these performance metrics; File S1: Guideline for Reporting Systematic Review; Table S1: Search Terms; Table S2: Characteristics of Included Studies; Table S3: Risk of Bias Assessment of Model Development with PROBAST + AI Development Tool; Table S4: Risk of Bias Assessment of Model Validation with PROBAST + AI Evaluation Tool.

## Abbreviations

The following abbreviations are used in this manuscript:

AI	Artificial Intelligence
AJCC	American Joint Committee on Cancer
AUC	Area Under the Receiver Operating Characteristic Curve
BNI	Bone Invasion
C-index	Concordance Index
CI	Confidence Interval
CSS	Cancer-Specific Survival
DFS	Disease-Free Survival
DSS	Disease-Specific Survival
ECE	Extranodal Extension
ML	Machine Learning
OCSCC	Oral Cavity Squamous Cell Carcinoma
OS	Overall Survival
PNI	Perineural Invasion
PRISMA	Preferred Reporting Items for Systematic Reviews and Meta-Analyses
PROBAST + AI	Prediction Model Risk of Bias Assessment Tool for Artificial Intelligence
RFS	Recurrence-Free Survival
ROC	Receiver Operating Characteristic
SVM	Support Vector Machine
TNM	Tumor, Node, Metastasis
LVI	Lymphovascular Invasion
CT	Computed Tomography
MRI	Magnetic Resonance Imaging
PET	Positron Emission Tomography
FDG-PET	Fluorodeoxyglucose Positron Emission Tomography
XGBoost	Extreme Gradient Boosting
MeSH	Medical Subject Headings
TRIPOD-AI	Transparent Reporting of a Multivariable Prediction Model for Individual Prognosis or Diagnosis–Artificial Intelligence Extension
