# Supplementary material for: Performance of Machine Learning Models for Prognosis Prediction in Oral Cavity Squamous Cell Carcinoma: A Systematic Review
Source: Cancers (Basel). 2026 Jul 14;18(14):2261. doi: 10.3390/cancers18142261 (PMC13406284; doi:10.3390/cancers18142261)
Supplement: Supplementary file 1 [file cancers-18-02261-s001.zip › cancers-4380914-supplementary.pdf]

cancers

MDPI

Supplemental Materials

Metric	Equation	How it is Calculated	What it Means	Range
**AUC** (Area Under the ROC Curve)	$AUC = \int_0^1 TPR(FPR)\, d(FPR)$	Compute the ROC curve by plotting True Positive Rate (TPR) vs. False Positive Rate (FPR) across all possible thresholds. Calculate the area under this curve.	Measures the ability of the model to discriminate between positive and negative classes across all thresholds. Higher values indicate better discrimination.	0.5 (no discrimination) to 1.0 (perfect discrimination)
**Accuracy** (Overall Accuracy)	$Accuracy = \frac{TP + TN}{TP + TN + FP + FN}$	Compute the proportion of all correct predictions (both positive and negative) out of the total number of predictions.	Indicates the overall correctness of the model. Can be influenced by class imbalance.	0 to 1 (1 = 100% accurate)
**Sensitivity** (Recall, True Positive Rate)	$Sensitivity = \frac{TP}{TP + FN}$	Compute the proportion of actual positives that are correctly identified by the model.	Measures the model's ability to identify positive cases (e.g., true events, disease). Higher values mean fewer missed positives.	0 to 1 (1 = perfect sensitivity)
**Specificity** (True Negative Rate)	$Specificity = \frac{TN}{TN + FP}$	Compute the proportion of actual negatives that are correctly identified by the model.	Measures the model's ability to identify negative cases. Higher values mean fewer false positives.	0 to 1 (1 = perfect specificity)
**C-index** (Concordance Index)	$C\text{-index} = \frac{\text{Number of Concordant Pairs}}{\text{Number of Comparable Pairs}}$	Evaluate all comparable pairs (i, j) where the observed time-to-event differs. The C-index is the proportion of pairs where the predicted risk correctly ranks the outcomes (higher risk → earlier event). Ties are counted as 0.5.	Measures the model's ability to correctly rank individuals by risk with respect to time-to-event data. Higher values indicate better predictive discrimination.	0.5 (no better than chance) to 1.0 (perfect concordance)

Supplementary Figure S1. Metric DefinitionsTPR represents true positive rate; FPR represents false positive rate; TP represents true positive; FP represents false positive; TN represents true negative; FN represents false negative.

Supplementary Table S1. Search Terms.

PubMed	("Squamous Cell Carcinoma of Head and Neck"[Mesh] OR "oral cavity squamous cell carcinoma" OR OCSCC OR "tongue squamous cell carcinoma" OR "mouth neoplas*" OR "oral cancer" OR "oral cavity cancer" OR "oral tongue carcinoma" OR "oral squamous cell carcinoma" OR "oral tongue squamous cell carcinoma" OR "floor of mouth cancer" OR "floor of mouth carcinoma" OR "mouth cancer" OR "buccal cancer" OR "tongue cancer" OR "buccal carcinoma" OR "mandibular cancer" OR "mandibular carcinoma" OR "alveolar ridge cancer" OR "alveolar ridge carcinoma" OR "retromolar trigone cancer" OR "retromolar trigone carcinoma" OR "hard palate cancer" OR "hard palate carcinoma") AND ("Machine Learning"[Mesh] OR "machine learning" OR "machine-learning" OR "deep learning" OR "deep-learning" OR "supervised learning" OR "unsupervised learning" OR "neural network*" OR "convolutional neural network*" OR "support vector machine*" OR "radiomic*" OR "feature selection" OR "LASSO" OR "penalized" OR "least absolute shrinkage" OR "survival prediction" OR "prediction model") AND ("nodal metastasis" OR "occult lymph node metastasis" OR "occult lymph node metastases" OR outcomes OR OS OR "overall survival" OR "overall-survival" OR EFS OR "event free survival" OR "event-free survival" OR DFS OR "disease free survival" OR "disease-free survival" OR PFS OR "progression free survival" OR "progression-free survival" OR TTP OR "time to progression" OR "time-to-progression" OR "time-to-event" OR recurren* OR RFS OR "recurrence free survival" OR "recurrence-free survival" OR "locoregional recurrence" OR mortality OR death)
Scopus	TITLE-ABS-KEY("oral cavity squamous cell carcinoma" OR OCSCC OR "tongue squamous cell carcinoma" OR "mouth neoplas*" OR "oral cancer" OR "oral cavity cancer"

	OR "oral squamous cell carcinoma" OR "oral tongue carcinoma" OR "oral tongue squamous cell carcinoma" OR "floor of mouth cancer" OR "floor of mouth carcinoma" OR "mouth cancer" OR "buccal cancer" OR "buccal carcinoma" OR "tongue cancer" OR "mandibular cancer" OR "mandibular carcinoma" OR "alveolar ridge cancer" OR "alveolar ridge carcinoma" OR "retromolar trigone cancer" OR "retromolar trigone carcinoma" OR "hard palate cancer" OR "hard palate carcinoma")<br>AND<br>TITLE-ABS-KEY("machine learning" OR "machine-learning" OR "deep learning" OR "deep-learning" OR "supervised learning" OR "unsupervised learning" OR "neural network*" OR "convolutional neural network*" OR "support vector machine*" OR radiomic* OR "feature selection" OR LASSO OR penalized OR "least absolute shrinkage" OR "survival prediction" OR "prediction model")<br>AND<br>TITLE-ABS-KEY("nodal metastasis" OR "occult lymph node metastasis" OR "occult lymph node metastases" OR outcomes OR OS OR "overall survival" OR "overall-survival" OR EFS OR "event free survival" OR "event-free survival" OR DFS OR "disease free survival" OR "disease-free survival" OR PFS OR "progression free survival" OR "progression-free survival" OR TTP OR "time to progression" OR "time-to-progression" OR "time-to-event" OR recurren* OR RFS OR "recurrence free survival" OR "recurrence-free survival" OR "locoregional recurrence" OR mortality OR death)
CINAHL	"oral cavity squamous cell carcinoma" OR OCSCC OR "tongue squamous cell carcinoma" OR "mouth neoplas*" OR "oral cancer" OR "oral cavity cancer" OR "oral squamous cell carcinoma" OR "oral tongue carcinoma" OR "oral tongue squamous cell carcinoma" OR "floor of mouth cancer" OR "floor of mouth carcinoma" OR "mouth cancer" OR "buccal cancer" OR "buccal carcinoma" OR "tongue cancer" OR "mandibular cancer" OR "mandibular carcinoma" OR "alveolar ridge cancer" OR "alveolar ridge carcinoma" OR "retromolar trigone cancer" OR "retromolar trigone carcinoma" OR "hard palate cancer" OR "hard palate carcinoma"<br>AND<br>"machine learning" OR "machine-learning" OR "deep learning" OR "deep-learning" OR "supervised learning" OR "unsupervised learning" OR "neural network*" OR "convolutional neural network*" OR "support vector machine*" OR radiomic* OR "feature selection" OR LASSO OR penalized OR "least absolute shrinkage" OR "survival prediction" OR "prediction model"<br>AND<br>"nodal metastasis" OR "occult lymph node metastasis" OR "occult lymph node metastases" outcomes OR OS OR "overall survival" OR "overall-survival" OR EFS OR "event free survival" OR "event-free survival" OR DFS OR "disease free survival" OR "disease-free survival" OR PFS OR "progression free survival" OR "progression-free survival" OR TTP OR "time to progression" OR "time-to-progression" OR "time-to-event" OR recurren* OR RFS OR "recurrence free survival" OR "recurrence-free survival" OR "locoregional recurrence" OR mortality OR death
Cochrane	("oral cavity squamous cell carcinoma" OR OCSCC OR "tongue squamous cell carcinoma" OR "mouth neoplas*" OR "oral cancer" OR "oral cavity cancer" OR "oral squamous cell carcinoma" OR "oral tongue carcinoma" OR "oral tongue squamous cell carcinoma" OR "floor of mouth cancer" OR "floor of mouth carcinoma" OR "mouth cancer" OR "buccal cancer" OR "buccal carcinoma" OR "tongue cancer" OR "mandibular cancer" OR "mandibular carcinoma" OR "alveolar ridge cancer" OR "alveolar ridge carcinoma" OR "retromolar trigone cancer" OR "retromolar trigone carcinoma" OR "hard palate cancer" OR "hard palate carcinoma")<br>AND<br>("machine learning" OR "machine-learning" OR "deep learning" OR "deep-learning" OR "supervised learning" OR "unsupervised learning" OR "neural network*" OR "convolutional neural network*" OR "support vector machine*" OR radiomic* OR "feature selection" OR LASSO OR penalized OR "least absolute shrinkage" OR "survival prediction" OR "prediction model")<br>AND<br>("nodal metastasis" OR "occult lymph node metastasis" OR "occult lymph node metastases" OR outcomes OR OS OR "overall survival" OR "overall-survival" OR EFS OR "event free survival" OR "event-free survival" OR DFS OR "disease free survival" OR "disease-free survival" OR PFS OR "progression free survival" OR "progression-free survival" OR TTP OR "time to progression" OR "time-to-progression" OR "time-to-event" OR recurren* OR RFS OR "recurrence free survival" OR "recurrence-free survival" OR "locoregional recurrence" OR mortality OR death)

Supplementary Table S2. Characteristics of Included Studies.

Study	Country	Data Source	Inputs	Model Algorithm	Outcome	Validation
Adachi 2024	Japan	Single institution	Digital Pathology, Pathologic	CLAM + SVM	Recurrence	Independent
Adeoye 2022	Hong Kong, China	Single institution	Clinical, Pathologic	DeepSurv	DSS/CSS, OS	Internal
Alabi 2023	USA	Registry/database	Clinical	Extreme random tree	OS	Internal
Alabi 2026	USA	Registry/database	Clinical	Voting ensemble	OS	Independent
Alabi 2021	USA	Registry/database	Clinical	Boosted Decision Tree	OS	Internal
Alabi 2020	Finland/Brazil	Multi-institution	Clinical, Pathologic	Boosted Decision Tree	Recurrence	Independent
Alabi 2019	Finland/Brazil	Multi-institution	Clinical, Pathologic	Artificial neural network	Recurrence	Internal
Alkhadar 2021	UK	Single institution	Clinical, Pathologic	Decision tree classifier	DFS/RFS	Independent
Bourdillon 2023	USA	Registry/database	Clinical, Pathologic	Decision tree classifier	Recurrence	Independent
Cai 2023	China	Single institution	Clinical, Pathologic, Immunologic	Multilayer perceptron	OS	Independent
Chen 2025	China	Single institution	Genomic/Molecular, Clinical	Random Forest	Recurrence	Independent
Chu 2020	Hong Kong, China	Single institution	Clinical, Pathologic	K-nearest neighbours	Progression	Internal
Csűry 2024	Germany	Single institution	Pathologic, Clinical	Naïve Bayes	DSS/CSS, Nodal Metastasis	Independent
Deepali 2025	India	Public dataset	Clinical, Genomic/Molecular	DeepOmicsSurv	OS	Independent
Diao 2021	China	Multi-institution	Immunologic	SVM	DFS/RFS, OS	Independent
Fatapour 2023	USA	Registry/database	Clinical	Gradient Boosting Machine	Recurrence	Independent
Fujima 2020	USA	Single institution	Radiologic	ResNet-101 deep learning classifier	DFS/RFS	Independent
Han 2025	China	Public dataset	Genomic/Molecular	Elastic Net	OS	Independent
Huang 2020	China	Public dataset	Genomic/Molecular, Immunologic	Random forest + SVM	OS	Internal
Huang 2025	Taiwan	Single institution	Clinical, Pathologic	XGBoost	Recurrence	Independent
Karadaghy 2019	USA	Registry/database	Clinical, Pathologic	Decision Forest	OS	Independent
Kim 2019	South Korea	Single institution	Pathologic	DeepSurv	OS	Independent

Lee 2025	Taiwan	Multi-institution	Clinical, Pathologic, Radiologic	Random Forest	OS	Independent
Li 2023	China	Single institution	Clinical, Immunologic	Light Gradient Boosting Machine	OS	Internal
Lin 2025	China	Registry/database	Clinical	DeepSurv-based deep neural network	OS	Internal
Lu 2017	USA	Single institution	Pathologic	Quadratic Discriminant Analysis	DSS/CSS	Internal
Mei 2025	China	Multi-institution	Clinical, Pathologic, Immunologic	K Neighbors Classifier	Progression	Independent
Mes 2020	Netherlands	Multi-institution	Radiologic, Clinical	Combined radiomic + clinical Cox model	OS, DFS/RFS	Independent
Nezamabadi Farahani 2024	Iran	Public dataset	Genomic/Molecular	Particle Swarm Optimization + Support Vector Regression	OS	Internal
Peng 2022	China	Registry/database	Clinical	Random Forest	DSS/CSS, OS	Independent
Pfaehler 2025	Germany	Single institution	Radiologic	Random Forest	Nodal Metastasis, Recurrence	Internal
Rosado 2013	Spain	Single institution	Clinical, Genomic/Molecular	SVM	OS	Internal
Salehi 2024	Sweden	Single institution	Clinical, Pathologic, Radiologic	Random Forest	Recurrence	Internal
Somyanonthanakul 2024	Thailand	Multi-institution	Clinical, Pathologic	Fuzzy Deep Learning	OS	Independent
Tan 2022	Hong Kong, China	Registry/database	Clinical	Random Forest classifier	OS	Internal
Tseng 2015	Taiwan	Registry/database	Clinical, Pathologic	Decision tree	DFS/RFS	Internal
Tseng 2015	Taiwan	Registry/database	Clinical, Pathologic	Decision tree	DSS/CSS	Internal
Vidiri 2025	Italy	Single institution	Clinical, Pathologic	Support Vector Machine	Recurrence	Independent
Vollmer 2024	USA	Public dataset	Clinical, Pathologic, Radiologic, Genomic/Molecular	Random Survival Forest	OS	Internal
Wang 2023	China	Registry/database	Clinical	Random Survival Forest	DSS/CSS	Internal

Supplementary Table S3. Risk of Bias Assessment of Model Development with PROBAST+AI Development Tool.

Study	Domain 1: Data					Domain 2: Predictors						Domain 3: Predictors						Domain 4: Analysis						OVERALL
	Q1	Q2	Q3	QA	AP	Q1	Q2	Q3	Q4	QA	AP	Q1	Q2	Q3	Q4	QA	AP	Q1	Q2	Q3	Q4	Q5	QA	
Adachi 2024	Y	PY	PY	L	L	Y	Y	PY	Y	L	L	Y	Y	PY	PY	L	L	PN	PY	NI	NA	PY	H	H
Adeoye 2022	Y	PY	PY	L	L	Y	Y	PY	Y	L	L	Y	Y	PY	Y	L	L	PY	Y	PY	NA	PY	L	L
Alabi 2023	Y	Y	PY	L	L	Y	Y	PY	Y	L	L	Y	Y	PY	Y	L	L	PN	PY	NI	NA	PY	H	H
Alabi 2026	Y	Y	PY	L	L	Y	Y	PY	Y	L	L	Y	Y	PY	Y	L	L	Y	Y	NI	NA	PY	L	L
Alabi 2021	Y	Y	PY	L	L	Y	Y	PY	Y	L	L	Y	Y	PY	Y	L	L	PY	Y	Y	Y	Y	L	L
Alabi 2020	Y	Y	Y	L	L	Y	Y	PY	Y	L	L	Y	Y	PY	Y	L	L	PY	Y	Y	Y	Y	L	L
Alabi 2019	Y	Y	PY	L	L	Y	Y	PY	Y	L	L	Y	Y	PY	Y	L	L	PN	PY	NI	NA	PY	H	H

https://doi.org/10.3390/xxxxx

Alkhadar 2021	Y	PY	PY	L	L	Y	Y	PY	Y	L	L	Y	Y	PY	Y	L	L	PN	PY	PY	NA	PY	H	H
Bourdillon 2023	Y	PY	PY	L	L	Y	Y	PY	Y	L	L	Y	Y	PY	Y	L	L	PY	Y	Y	NA	PY	L	L
Cai 2023	Y	Y	Y	L	L	Y	Y	PY	Y	L	L	Y	Y	PY	Y	L	L	PY	Y	Y	NA	PY	L	L
Chen 2025	Y	Y	Y	L	L	Y	Y	Y	Y	L	L	Y	Y	Y	Y	L	L	PY	Y	PY	NA	PY	L	L
Chu 2020	Y	Y	PY	L	L	Y	Y	PY	Y	L	L	Y	Y	PY	Y	L	L	PN	PY	PY	NA	PY	H	H
Csury 2024	Y	Y	PY	L	L	Y	Y	PY	Y	L	L	Y	Y	PY	Y	L	L	PY	PY	PY	NA	PY	L	L
Deepali 2025	PY	PY	PY	L	L	Y	Y	PY	Y	L	L	Y	Y	PY	Y	L	L	PN	PY	PY	NA	PY	H	H
Diao 2021	Y	Y	Y	L	L	Y	Y	PY	Y	L	L	Y	Y	PY	Y	L	L	PY	Y	Y	NA	PY	L	L
Fatapour 2023	PY	PY	PY	L	L	Y	Y	PY	Y	L	L	Y	Y	PY	Y	L	L	PN	PY	PY	NA	PY	H	H
Fujima 2020	Y	PY	PY	L	L	Y	Y	PY	Y	L	L	Y	Y	PY	Y	L	L	PN	PY	PY	NA	PY	H	H
Han 2025	PY	PY	PY	L	L	Y	Y	PY	Y	L	L	Y	Y	PY	Y	L	L	PN	PY	PY	NA	PY	H	H
Huang 2020	Y	Y	PY	L	L	Y	Y	PY	Y	L	L	Y	Y	PY	Y	L	L	PY	PY	PY	NA	PY	L	L
Huang 2025	PY	PY	PY	L	L	Y	Y	PY	Y	L	L	Y	Y	PY	Y	L	L	PN	PY	PY	NA	PY	H	H
Karadaghy 2019	Y	Y	PY	L	L	Y	Y	PY	Y	L	L	Y	Y	PY	Y	L	L	PY	PY	PY	NA	PY	L	L
Kim 2019	PY	PY	PY	L	L	Y	Y	PY	Y	L	L	Y	Y	PY	Y	L	L	PN	PY	PY	NA	PY	H	H
Lee 2025	PY	PY	PY	L	L	Y	Y	PY	Y	L	L	Y	Y	PY	Y	L	L	PN	PY	PY	NA	PY	H	H
Li 2023	PY	PY	PY	L	L	Y	Y	PY	Y	L	L	Y	Y	PY	Y	L	L	PN	PY	PY	NA	PY	H	H
Lin 2025	PY	PY	PY	L	L	Y	Y	PY	Y	L	L	Y	Y	PY	Y	L	L	PN	PY	PY	NA	PY	H	H
Lu 2017	Y	Y	PY	L	L	Y	Y	PY	Y	L	L	Y	Y	PY	Y	L	L	PY	PY	PY	NA	PY	L	L
Mei 2025	PY	PY	PY	L	L	Y	Y	PY	Y	L	L	Y	Y	PY	Y	L	L	PN	PY	PY	NA	PY	H	H
Mes 2020	Y	Y	PY	L	L	Y	Y	PY	Y	L	L	Y	Y	PY	Y	L	L	PY	PY	PY	NA	PY	L	L
Nezamabadi Farahani 2024	PY	PY	PY	L	L	Y	Y	PY	Y	L	L	Y	Y	PY	Y	L	L	PN	PY	PY	NA	PY	H	H
Peng 2022	Y	Y	PY	L	L	Y	Y	PY	Y	L	L	Y	Y	PY	Y	L	L	PY	PY	PY	NA	PY	L	L
Pfaehler 2025	Y	PY	PY	L	L	Y	Y	PY	Y	L	L	Y	Y	PY	Y	L	L	PN	PY	PY	NA	PY	H	H
Rosado 2013	PY	PY	PY	L	L	Y	Y	PY	Y	L	L	Y	Y	PY	Y	L	L	PN	PN	NI	NA	PN	H	H
Salehi 2024	PY	PY	PY	L	L	Y	Y	PY	Y	L	L	Y	Y	PY	Y	L	L	PN	PY	PY	NA	PY	H	H
Somyanon-thanakul 2024	PY	PY	PY	L	L	Y	Y	PY	Y	L	L	Y	Y	PY	Y	L	L	PN	PY	NI	NA	PN	H	H
Tan 2022	Y	Y	PY	L	L	Y	Y	PY	Y	L	L	Y	Y	PY	Y	L	L	PY	Y	Y	Y	Y	L	L
Tseng 2015	PY	PY	PY	L	L	Y	Y	PY	Y	L	L	Y	Y	PY	Y	L	L	PN	PN	NI	NA	PN	H	H
Tseng 2020	Y	Y	PY	L	L	Y	Y	PY	Y	L	L	Y	Y	PY	Y	L	L	PY	Y	Y	NA	Y	L	L
Vidiri 2025	PY	PY	PY	L	L	Y	Y	PY	Y	L	L	Y	Y	PY	Y	L	L	PN	PY	PY	NA	PY	H	H
Vollmer 2024	PY	PY	PY	L	L	Y	Y	PY	Y	L	L	Y	Y	PY	Y	L	L	PN	PY	PY	NA	PY	H	H
Wang 2023	Y	Y	Y	L	L	Y	Y	PY	Y	L	L	Y	Y	PY	Y	L	L	Y	Y	Y	NA	Y	L	L

Supplementary Table S4. Risk of Bias Assessment of Model Validation with PROBAST+AI Evaluation Tool.

Study	Domain 1: Data					Domain 2: Predictors						Domain 3: Predictors						Domain 4: Analysis								
	Q1	Q2	Q3	QA	AP	Q1	Q2	Q3	Q4	QA	AP	Q1	Q2	Q3	Q4	QA	AP	Q1	Q2	Q3	Q4	Q5	Q6	Q7	QA	OVERALL
Adachi 2024	PY	PY	PY	L	L	Y	Y	PY	Y	L	L	Y	Y	PY	PY	L	L	PN	PY	NI	NA	PY	N	N	H	H
Adeoye 2022	PY	PY	PY	L	L	Y	Y	PY	Y	L	L	Y	Y	PY	Y	L	L	PY	Y	PY	NA	PY	L	L	L	L
Alabi 2023	PY	PY	PY	L	L	Y	Y	PY	Y	L	L	Y	Y	PY	Y	L	L	PN	PY	NI	NA	PY	H	H	H	H
Alabi 2026	PY	PY	PY	L	L	Y	Y	PY	Y	L	L	Y	Y	PY	Y	L	L	PY	Y	NI	NA	PY	L	L	L	L
Alabi 2021	PY	PY	PY	L	L	Y	Y	PY	Y	L	L	Y	Y	PY	Y	L	L	PY	Y	Y	Y	Y	L	L	L	L
Alabi 2020	PY	PY	PY	L	L	Y	Y	PY	Y	L	L	Y	Y	PY	Y	L	L	PY	Y	Y	Y	Y	L	L	L	L
Alabi 2019	PY	PY	PY	L	L	Y	Y	PY	Y	L	L	Y	Y	PY	Y	L	L	PN	PY	NI	NA	PY	H	H	H	H
Alkhadar 2021	NA	NA	NA	NA	NA	NA	NA	NA	NA	NA	NA	NA	NA	NA	NA	NA	NA	NA	NA	NA	NA	NA	NA	NA	H	H
Bourdillon 2023	PY	PY	PY	L	L	Y	Y	PY	Y	L	L	Y	Y	PY	Y	L	L	PY	Y	Y	NA	PY	L	L	L	L
Cai 2023	PY	PY	PY	L	L	Y	Y	PY	Y	L	L	Y	Y	PY	Y	L	L	PY	Y	Y	NA	PY	L	L	L	L
Chen 2025	PY	PY	PY	NA	PY	L	L	Y	Y	Y	Y	L	L	Y	Y	Y	Y	L	L	PY	Y	PY	NA	PY	L	L
Chu 2020	PY	PY	PY	NA	PY	L	L	Y	Y	PY	Y	L	L	Y	Y	PY	Y	L	L	PN	PY	PY	NA	PY	H	H
Csury 2024	PY	PY	PY	NA	PY	L	L	Y	Y	PY	Y	L	L	Y	Y	PY	Y	L	L	PY	PY	PY	NA	PY	L	L

Deepali 2025	PN	PY	PY	NA	PY	H	H	Y	Y	PY	Y	L	L	Y	Y	PY	Y	L	L	PN	PY	PY	NA	PY	H	H
Diao 2021	PY	PY	PY	NA	PY	L	L	Y	Y	PY	Y	L	L	Y	Y	PY	Y	L	L	PY	Y	Y	NA	PY	L	L
Fatapour 2023	PN	PY	PY	NA	PY	H	H	Y	Y	PY	Y	L	L	Y	Y	PY	Y	L	L	PN	PY	PY	NA	PY	H	H
Fujima 2020	PY	PY	PY	NA	PY	L	L	Y	Y	PY	Y	L	L	Y	Y	PY	Y	L	L	PN	PY	PY	NA	PY	H	H
Han 2025	PN	PY	PY	NA	PY	H	H	Y	Y	PY	Y	L	L	Y	Y	PY	Y	L	L	PN	PY	PY	NA	PY	H	H
Huang 2020	PY	PY	PY	NA	PY	L	L	Y	Y	PY	Y	L	L	Y	Y	PY	Y	L	L	PY	PY	PY	NA	PY	L	L
Huang 2025	PN	PY	PY	NA	PY	H	H	Y	Y	PY	Y	L	L	Y	Y	PY	Y	L	L	PN	PY	PY	NA	PY	H	H
Karadaghy 2019	PY	PY	PY	NA	PY	L	L	Y	Y	PY	Y	L	L	Y	Y	PY	Y	L	L	PY	PY	PY	NA	PY	L	L
Kim 2019	PN	PY	PY	NA	PY	H	H	Y	Y	PY	Y	L	L	Y	Y	PY	Y	L	L	PN	PY	PY	NA	PY	H	H
Lee 2025	PN	PY	PY	NA	PY	H	H	Y	Y	PY	Y	L	L	Y	Y	PY	Y	L	L	PN	PY	PY	NA	PY	H	H
Li 2023	PN	PY	PY	NA	PY	H	H	Y	Y	PY	Y	L	L	Y	Y	PY	Y	L	L	PN	PY	PY	NA	PY	H	H
Lin 2025	PN	PY	PY	NA	PY	H	H	Y	Y	PY	Y	L	L	Y	Y	PY	Y	L	L	PN	PY	PY	NA	PY	H	H
Lu 2017	PY	PY	PY	NA	PY	L	L	Y	Y	PY	Y	L	L	Y	Y	PY	Y	L	L	PY	PY	PY	NA	PY	L	L
Mei 2025	PN	PY	PY	NA	PY	H	H	Y	Y	PY	Y	L	L	Y	Y	PY	Y	L	L	PN	PY	PY	NA	PY	H	H
Mes 2020	PY	PY	PY	NA	PY	L	L	Y	Y	PY	Y	L	L	Y	Y	PY	Y	L	L	PY	PY	PY	NA	PY	L	L
Nezamabadi Farahani 2024	PN	PY	PY	NA	PY	H	H	Y	Y	PY	Y	L	L	Y	Y	PY	Y	L	L	PN	PY	PY	NA	PY	H	H
Peng 2022	PY	PY	PY	NA	PY	L	L	Y	Y	PY	Y	L	L	Y	Y	PY	Y	L	L	PY	PY	PY	NA	PY	L	L
Pfaehler 2025	PN	PY	PY	NA	PY	H	H	Y	Y	PY	Y	L	L	Y	Y	PY	Y	L	L	PN	PY	PY	NA	PY	H	H
Rosado 2013	NA	NA	NA	NA	NA	NA	NA	NA	NA	NA	NA	NA	NA	NA	NA	NA	NA	NA	NA	NA	NA	NA	NA	NA	H	H
Salehi 2024	PN	PY	PY	NA	PY	H	H	Y	Y	PY	Y	L	L	Y	Y	PY	Y	L	L	PN	PY	PY	NA	PY	H	H
Somyanonthakul 2024	PN	PY	PY	NA	PN	H	H	Y	Y	PY	Y	L	L	Y	Y	PY	Y	L	L	PN	PY	NI	NA	PN	H	H
Tan 2022	PY	PY	PY	Y	Y	L	L	Y	Y	PY	Y	L	L	Y	Y	PY	Y	L	L	PY	Y	Y	Y	Y	L	L
Tseng 2015	NA	NA	NA	NA	NA	NA	NA	NA	NA	NA	NA	NA	NA	NA	NA	NA	NA	NA	NA	NA	NA	NA	NA	NA	H	H
Tseng 2020	PY	PY	PY	NA	Y	L	L	Y	Y	PY	Y	L	L	Y	Y	PY	Y	L	L	PY	Y	Y	NA	Y	L	L
Vidiri 2025	PN	PY	PY	NA	PY	H	H	Y	Y	PY	Y	L	L	Y	Y	PY	Y	L	L	PN	PY	PY	NA	PY	H	H
Vollmer 2024	PN	PY	PY	NA	PY	H	H	Y	Y	PY	Y	L	L	Y	Y	PY	Y	L	L	PN	PY	PY	NA	PY	H	H
Wang 2023	PY	PY	PY	NA	Y	L	L	Y	Y	PY	Y	L	L	Y	Y	PY	Y	L	L	Y	Y	Y	NA	Y	L	L
